# Glucose variability and ICU outcome

**DOI:** 10.1186/cc9822

**Published:** 2011-03-11

**Authors:** S Todi, M Bhattacharyya

**Affiliations:** 1 AMRI Hospitals, Kolkata, India

## Introduction

Glycemic excursion or glucose variability (GV) was explored recently as a contributor of mortality, when studies concentrating on strict blood sugar control failed to show consistent results. The objective of this study was to determine the implication of glucose variability on ICU mortality in a heterogeneous ICU population.

## Methods

The study was conducted in a medical/surgical ICU (45 beds) in a private teaching tertiary care hospital in India. A nurse-driven subcutaneous and intravenous insulin protocol (modified Yale) was followed for sugar control with a target CBG of ≤150 mg/dl. Blood sugar was checked as per patient requirement, both by point-of care-testing and central laboratory. The outcome measure was ICU mortality. From the prospectively collected glucose values, mean blood glucose (MBG) was measured for each patient and glycemic variability (GV) calculated as the standard deviation (SD) and glycemic lability index of MBG. GV was correlated with mortality.

## Results

**Figure 1:**
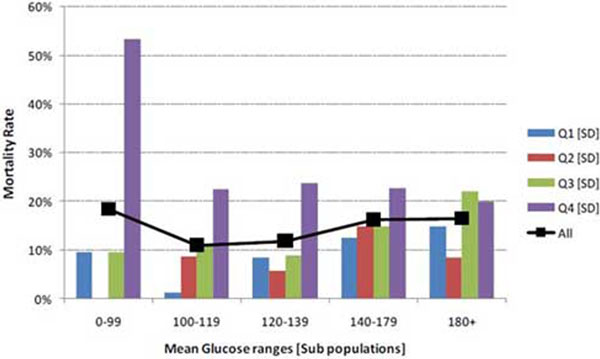
**Mortality rates in quartile ranges of SD within subpopulations**.

The study was conducted from January 2009 until November 2009. All consecutive patients with four or more blood sugar measurements were considered. A total of 11,335 blood sugar records were analyzed from 2,208 patients during this time. The mean age of the study population was 61 (SD ± 16.71). In total, 58.96% were male and 77.8% were medical admissions. Mean APACHE IV score was 56.9. MBG of the study population was divided into five subgroups. Each subgroup had four quartiles of rising SD along with mortality. Mortality was higher in the highest quartiles of SD in each of five subgroups of patients. Mortality was highest in the subgroup with lowest range of MBG and who had maximum variability or highest SD. In our study cohort, 212 patients (9.6%) had hypoglycemia. In this cohort also mortality increased from 6, 10, 11, 16%, respectively, with rising SD in the same way as the whole cohort. See Figure 1.

## Conclusions

In summary, this study demonstrated that glucose variability is associated with ICU mortality in a large heterogeneous cohort of ICU patients. This effect was particularly strong among patients in the euglycemic range.

